# DETC Induces *Leishmania* Parasite Killing in Human *In Vitro* and Murine *In Vivo* Models: A Promising Therapeutic Alternative in Leishmaniasis

**DOI:** 10.1371/journal.pone.0014394

**Published:** 2010-12-21

**Authors:** Ricardo Khouri, Fernanda Novais, Gisélia Santana, Camila Indiani de Oliveira, Marcos André Vannier dos Santos, Aldina Barral, Manoel Barral-Netto, Johan Van Weyenbergh

**Affiliations:** 1 LIMI, LIP, LBP, Centro de Pesquisa Gonçalo Moniz, Fundação Oswaldo Cruz (FIOCRUZ), Salvador-Bahia, Brazil; 2 Institute for Investigation in Immunology (iii), INCT, São Paulo, Brazil; State University of Campinas (UNICAMP), Brazil

## Abstract

**Background:**

Chemotherapy remains the primary tool for treatment and control of human leishmaniasis. However, currently available drugs present serious problems regarding side-effects, variable efficacy, and cost. Affordable and less toxic drugs are urgently needed for leishmaniasis.

**Methodology/Principal Findings:**

We demonstrate, by microscopy and viability assays, that superoxide dismutase inhibitor diethyldithiocarbamate (DETC) dose-dependently induces parasite killing (*p*<0.001) and is able to “sterilize” *Leishmania amazonensis* infection at 2 mM in human macrophages *in vitro*. We also show that DETC-induced superoxide production (*p*<0.001) and parasite destruction (*p*<0.05) were reverted by the addition of the antioxidant N-acetylcysteine, indicating that DETC-induced killing occurs through oxidative damage. Furthermore, ultrastructural analysis by electron microscopy demonstrates a rapid and highly selective destruction of amastigotes in the phagosome upon DETC treatment, without any apparent damage to the host cell, including its mitochondria. In addition, DETC significantly induced parasite killing in *Leishmania* promastigotes in axenic culture. In murine macrophages infected with *Leishmania braziliensis*, DETC significantly induced *in vitro* superoxide production (*p* = 0.0049) and parasite killing (*p* = 0.0043). *In vivo* treatment with DETC in BALB/C mice infected with *Leishmania braziliensis* caused a significant decrease in lesion size (*p*<0.0001), paralleled by a 100-fold decrease (*p* = 0.0087) in parasite burden.

**Conclusions/Significance:**

Due to its strong leishmanicidal effect in human macrophages *in vitro*, its *in vivo* effectiveness in a murine model, and its previously demonstrated *in vivo* safety profile in HIV treatment, DETC treatment might be considered as a valuable therapeutic option in human leishmaniasis, including HIV/*Leishmania* co-infection.

## Introduction

Leishmaniasis is endemic in several parts of the world, with a global prevalence of over 12 million cases. Divided in two main groups, leishmaniasis can affect the skin (cutaneous leishmaniasis) or viscera (visceral leishmaniasis). There are 1,500,000 new cases of cutaneous leishmaniasis emerging every year [1]–[4]. The infection is caused by protozoan parasites of the genus *Leishmania*, transmitted by the bite of the female sand fly vector. Several *Leishmania* species are able to cause a wide spectrum of clinical manifestations of cutaneous leishmaniasis, ranging from the mild cutaneous form (localized cutaneous leishmaniasis; LCL), multiple non-ulcerative nodules (diffuse cutaneous leishmaniasis; DCL) and the disfiguring mucosal form (mucocutaneous leishmaniasis; MCL). In Brazil, *Leishmania (L.) braziliensis* causes LCL and MCL, whereas *L. amazonensis* causes LCL and, sporadically, DCL [1]–[5]. New World LCL is not life-threatening, but there is a marked variability in healing time, and an increasing frequency of therapeutic failure [6]–[7]. DCL and MCL are disfiguring and possibly life-threatening forms of the disease, if not properly treated. Standard chemotherapy (pentavalent antimonial - Sb^v^) leads to the resolution of the disease and thus avoids parasite dissemination and lifelong cutaneous scars in LCL and MCL, but no effective treatment has been described for DCL, being refractory to currently available treatment [7]. Pentavalent antimonials and amphotericin B are today's first and second choice, respectively, to treat cutaneous leishmaniasis. However, these drugs present serious problems regarding side-effects, variable efficacy and are expensive [4], [8]–[9]. Recently, our group has shown the importance of superoxide dismutase 1 (CuZnSOD/SOD1) in the control of parasite survival *in vitro*. The use of diethyldithiocarbamate (DETC), a copper-chelating compound that inhibits SOD1 [10], strongly antagonized the effect of IFN-β in infected human macrophages [11]. In addition, SOD1 was expressed in situ in biopsies of cutaneous leishmaniasis lesions [11] and *ex vivo* SOD1 plasma levels predict therapeutic failure in cutaneous leishmaniasis patients, (Khouri *et al.*, submitted). In the present work, we further investigated the effect of DETC *in vitro* and *in vivo*. DETC was non-toxic in human macrophages and PBMC at the concentration that induced strong leishmanicidal activity in intracellular parasite forms. This leishmanicidal activity was superoxide-dependent and reverted by the antioxidant N-acetylcysteine (NAC). By electron microscopy, selective intracellular destruction of amastigotes was documented inside the phagosome, without any damage to the host cell, including mitochondria. DETC treatment was also effective *in vivo*, causing a 100-fold decrease in parasite load in a murine model of *Leishmania braziliensis* infection, confirming its therapeutic potential.

## Methods

### Ethics statement

Balb/c Mice were used at 6 to 8 weeks of age. Animal husbandry, experimentation and welfare in our facility complies with the International Guiding Principles for Biomedical Research Involving Animals and is approved by the Animal Care Ethics Committee from CPqGM/FIOCRUZ.

### Reagents

All chemicals were purchased from Sigma, cell culture media and sera were obtained from Invitrogen Life Science, endotoxin-free sterile disposables were used in all experiments.

### Human macrophage culture and infection

Briefly, human monocytes were isolated from peripheral blood mononuclear cells (PBMC) of healthy donors through Ficoll gradient centrifugation and plastic adherence, and differentiated *in vitro* into macrophages (7 days). Human monocytes and macrophages were cultivated in RPMI medium or DMEM medium supplemented with 5% human AB serum. Macrophages were infected (5∶1) with *Leishmania (L.) amazonensis* (MHOM/BR/87/BA125) for 4 h and treated for 48 h with diethyldithiocarbamate (DETC, CuZn superoxide dismutase/SOD1 inhibitor), in the presence or absence of NAC (N-acetylcysteine).

### Viability, apoptosis and necrosis assay

For cell viability, PBMC were seeded in 24-well tissue culture plates at a density of 1×10^6^ cells per well. Twenty four hours later, cells were stained with trypan blue and viable cells were counted using optical microscopy. For apoptosis and necrosis, both annexinV-binding assay and Hoechst 33342 assay were used.

### Murine macrophage culture and infection

Resident macrophages were obtained after peritoneal injection of 5 ml of RPMI in BALB/c mice. Peritoneal exudate cells (3×10^5^cells) were plated onto glass coverslips placed within the wells of a 24-well plate containing complete culture medium (RPMI medium or DMEM medium supplemented with 10% fetal calf serum (FCS)). Non-adherent cells were washed out and murine macrophages were cultivated in complete culture medium. Macrophages were infected with *L. amazonensis* (MHOM/BR/87/BA125) or *L. braziliensis* (MHOM/BR/01/BA788) for 4 and 24 hours, respectively, and treated with diethyldithiocarbamate (DETC, CuZn superoxide dismutase/SOD1 inhibitor) for 48 h.

### Parasite culture

*L. amazonensis* (MHOM/BR/87/BA125) and *L. braziliensis* (MHOM/BR/01/BA788) strain cultures were maintained *in vitro* as proliferating promastigotes in Schneider's insect medium, supplemented with 10% FCS and 40 µg/ml of gentamycin.

### Quantification of parasite burden

After 48 h of treatment, macrophage monolayers were washed and stained with hematoxylin/eosin and the number of amastigotes per 100 cells were counted by optical microscopy (duplicates for each sample). Similar results were obtained with macrophages directly cultivated upon glass cover slips or macrophages removed by gentle scraping and deposited on glass slides in a cytocentrifuge (Cytospin).

### Parasite Survival

After 48 h of infected macrophage treatment, cell monolayers were washed, and medium was replaced by 0.5 ml of Schneider's medium, supplemented with 10% FCS. Cultures were maintained at 23°C for 8 additional days. Intracellular survival of *L. amazonensis* amastigotes was quantified by counting proliferating extracellular motile promastigotes, as previously described [11].

### Quantification of Superoxide

Superoxide production was quantified by two techniques: 1) by adding hydroxylamine (0.5 mM) [12] during cell culture, which converts superoxide into nitrite plus nitrate, and which was reduced by VCl_3_ [13] and quantified using Griess reagent; and 2) by hydroethidine (Invitrogen) staining and quantification of mean fluorescence intensity (MFI) cells by flow cytometry (FACSort; BD Biosciences) [11].

### Transmission electron microscopy

Cells were fixed in a solution of 2.5% glutaraldehyde grade II, 2% formaldehyde and 2.5 mM CaCl_2_ in 0.1 M sodium cacodylate buffer pH 7.2, post-fixed in 1% osmium tetroxide and 0.8% potassium ferricyanide in the same buffer, dehydrated in an acetone series and embedded in Polybed resin. Thin sections were stained with uranyl acetate and lead citrate and observed under a Zeiss 109 transmission electron microscope.

### *In vivo* infection in a murine model

A murine model closely resembling human pathology was previously described [14]. Briefly, female BALB/c mice were obtained from the Centro de Pesquisas Gonçalo Moniz/Fundação Oswaldo Cruz Animal Facility, where they were maintained under specific pathogen-free conditions. Stationary-phase promastigotes (10^5^ parasites in 10 µl of saline) of *L. braziliensis* strain (MHOM/BR/01/BA788) were inoculated into the left ear dermis of age-matched BALB/c mice using a 27.5-gauge needle. After the second week post-infection, a group of mice (n = 10) was treated daily with intraperitoneal injections of DETC (50 mg/kg diluted in saline), a control group was treated with intraperitoneal injections of saline (n = 10). Lesion size was monitored weekly for 10 weeks using a digital caliper (Thomas Scientific, Swedesboro, NJ).

### Parasite load quantification

Parasite load was determined using a quantitative limiting-dilution assay as described previously [15]. Briefly, infected ears and retromaxillar draining lymph nodes (LNs) were aseptically excised at 2, 5, and 10 weeks postinfection and homogenized in Schneider medium (Sigma Chemical Co., St. Louis, MO). The homogenates were serially diluted in Schneider medium with 10% FCS and seeded into 96-well plates containing biphasic blood agar (Novy-Nicolle-McNeal) medium. The number of viable parasites was determined from the highest dilution at which promastigotes could be grown after up to 2 weeks of incubation at 25°C.

### Statistical analysis

According to normality analysis (Kolmogorov-Smirnov), parametric (Student's *t* test, one-way ANOVA with Bonferroni's multiple test correction or post-test for linear trend and Area Under Curve with Student's *t* test) and non-parametric tests (Mann Whitney test), all two-tailed, were performed using GraphPad 5.0 software. Data are presented as mean ± SEM, and differences were considered significant at p<0.05.

## Results

### Dose-dependent leishmanicidal effect of DETC in *Leishmania*-infected human macrophages

**Figure 1:**
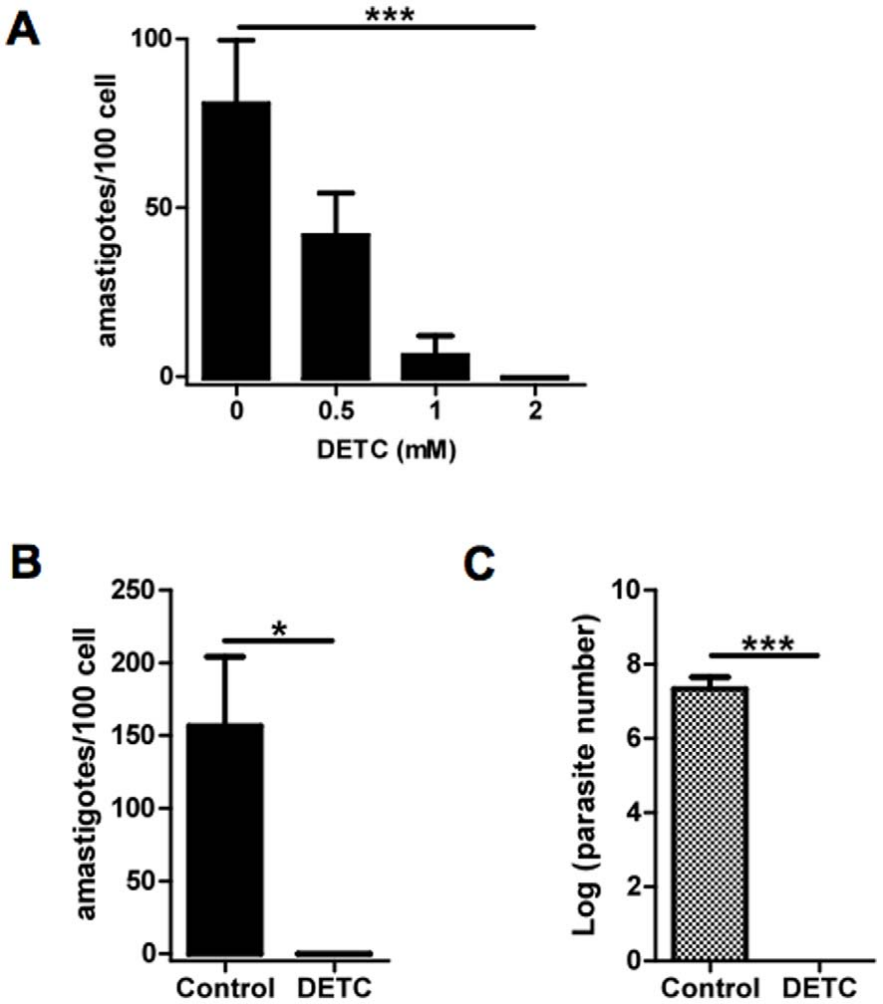
Dose-dependent leishmanicidal effect of DETC in *Leishmania*-infected human macrophages. Human monocyte-derived macrophages were infected with *Leishmania amazonensis* promastigotes (5∶1 ratio). (A) After infection, cells were treated with increasing concentrations of DETC and the number of intracellular amastigotes (symbols) was quantified as described in Material and Methods. Each bar represents the mean ± SEM of 100 cells counted in duplicate (One-way ANOVA, ***p = 0.0001; post test for linear trend, ***p<0.001) representative of two different donors. (B) and (C) *Leishmania amazonensis*-infected macrophages were treated 48 h after infection with 2 mM of DETC. (B) The number of intracellular amastigotes was quantified as described in Material and Methods. Results are expressed as number of amastigotes/100 cells. Each bar represents the mean ± SEM of 4 donors (Paired *t* test, *p = 0.046). (C) Intracellular survival of *Leishmania amazonensis* amastigotes was quantified by transformation of proliferating extracellular motile promastigotes in Schneider's medium. Each bar represents the mean ± SEM of 4 donors (Paired *t* test, ***p = 0.0002).

We investigated the effect of DETC upon parasite burden of *L. amazonensis* infected human macrophages. As shown in Figure 1A, the effect of DETC upon parasite load was dose-dependent, being effective at 0.5 mM and 1 mM (IC 50) and optimal at 2 mM (no surviving parasites detected) (One-way ANOVA, ***p = 0.0001; post test for linear trend, ***p<0.001) (Fig. 1A). At concentrations below 10 mM, no toxicity was observed in PBMC tested as evidenced by Hoechst 33342, annexinV/PI staining and trypan blue permeability (data not shown). The 2 mM concentration of DETC was used in all further experiments, and was able to “sterilize” parasite load at 48 h in 4 donors tested (Paired *t* test, *p = 0.046) (Fig. 1B). To confirm the apparent sterilizing effect of DETC upon parasite burden, intracellular parasite survival was quantified by transformation of amastigotes into proliferating extracellular motile promastigotes in Schneider's medium (10). Indeed, viability of recovered amastigotes from DETC-treated culture was consistenly zero (n = 4), whereas amastigotes recovered from control cultures were efficiently transformed into exponentially growing promastigotes (Paired *t* test, ***p = 0.0002) (Fig. 1C).

### SOD inhibitor DETC and antioxidant (NAC) reciprocally regulate parasite killing in *Leishmania*-infected human macrophages

**Figure 2:**
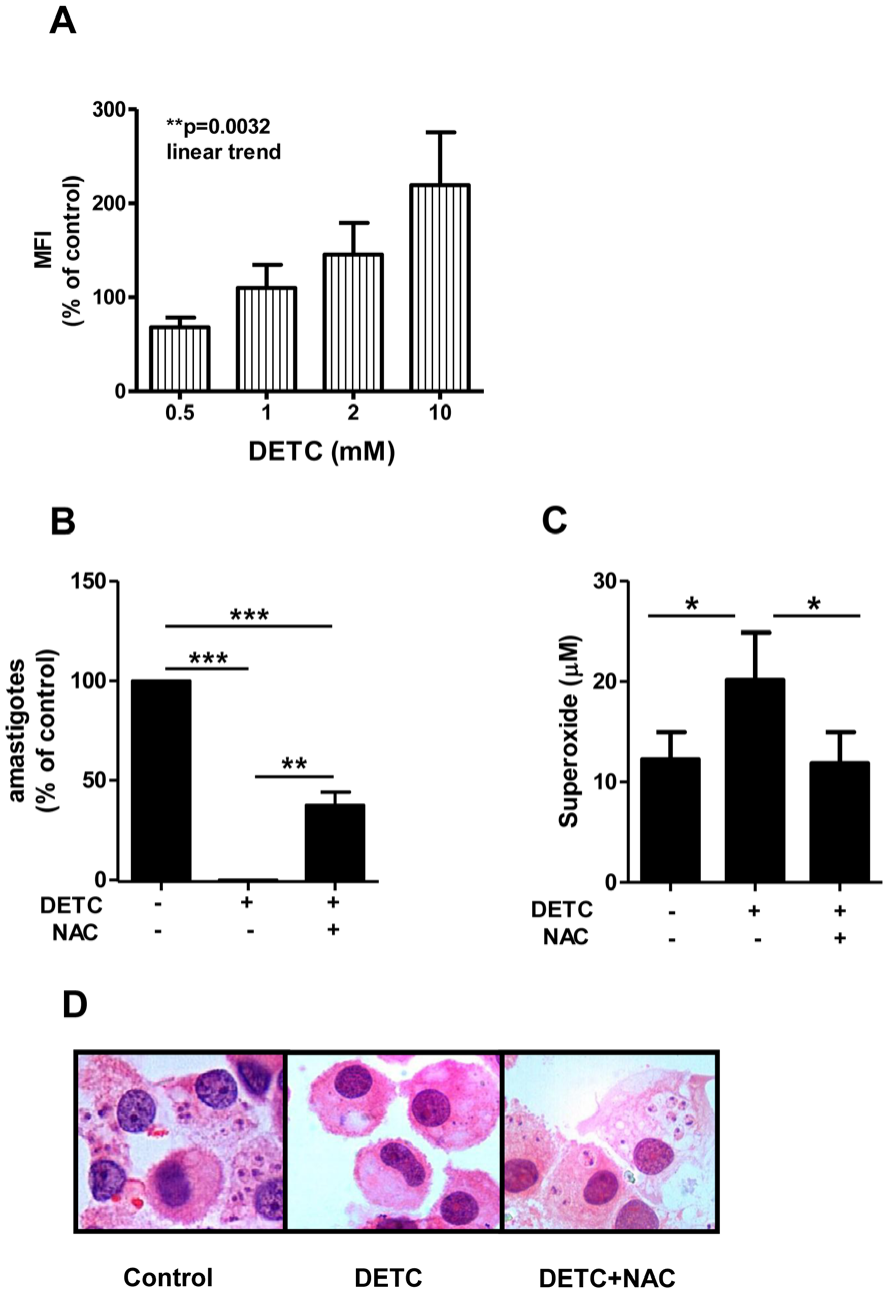
DETC leishmanicidal activity in human macrophages is reverted by antioxidant treatment. (A) Uninfected PBMC from normal donors were triggered with PMA (100 ng/ml), treated with increasing doses of DETC and stained with hydroethidine for 30 minutes. Superoxide production was measured by flow cytometry (Mean Fluorescence Intensity - MFI). Monocytes were gated from the whole PBMC population based on size and granularity and analysed separately for superoxide production. Each bar represents the mean ± SEM of 3 donors. (Repeated Measures ANOVA, *p = 0.018; post-test for linear trend, **p<0.0032). (B, C, and D) *Leishmania amazonensis*-infected human macrophages were treated with SOD inhibitor DETC (2 mM) in the absence or presence of antioxidant NAC (10 mM). (B) Hydroxylamine (0.5 mM) was added to the cultures and supernatant was collected at 48 h. Superoxide production was quantified as described in Materials and Methods. Each bar represents the mean ± SEM of 3 donors (Repeated Measures ANOVA, ***p = 0.0001; Bonferroni's correction, ***p<0.001, **p<0.01). (C) Cells were harvested at 48 h and the number of intracellular amastigotes was quantified as described in Materials and Methods. Each bar represents the mean ± SEM of 3 donors (Repeated Measures ANOVA, *p = 0.011; Bonferroni's correction, *p<0.05). (D) Cells were fixed on glass slides, stained with hematoxylin and eosin, and photographed (1000× magnification).

To test the hypothesis that DETC's leishmanicidal effect was due to an increase of superoxide release, superoxide production was monitored using a superoxide-specific fluorescent probe (hydroethidine) in PBMC from normal donors, triggered with PMA and treated with increasing doses of DETC for 30 minutes. DETC induced a significant dose-dependent increase in intracellular superoxide in monocytes (Repeated Measures ANOVA, *p = 0.018; post-test for linear trend, **p<0.0032) (Fig. 2A) but not in lymphocytes (Repeated Measures ANOVA, p = 0.15, data not shown). Interestingly, there was a peak of superoxide production in both monocyte and lymphocyte subpopulations treated with 10 mM of DETC (Fig. 2A and data not shown), coinciding with the induction of apoptosis in PBMC (data not shown). Next, the effect of antioxidant NAC (a Glutathione precursor) was used to verify superoxide-dependent parasite killing in DETC-treated human macrophages infected with *Leishmania*. As shown in figure 2B, DETC increased superoxide production by 64%, which was completely reversed by NAC (Repeated Measures ANOVA, ***p = 0.0001; Bonferroni's correction, ***p<0.001, **p<0.01). DETC-induced killing was largely (63%) inhibited by NAC (Repeated Measures ANOVA, *p = 0.011; Bonferroni's correction, *p<0.05) (Fig. 2C), suggesting that DETC-induced killing occurs, at least partly, through oxidative damage. Since DETC treatment lead to complete clearance of parasites without any apparent cytotoxicity, as evidenced by intact cell morphology (Fig. 2D), the damage caused by increasing free radicals seemed to be selectively directed towards intracellular parasites.

### Selective toxicity towards intracellular amastigotes in DETC-treated *Leishmania*-infected human macrophages

**Figure 3:**
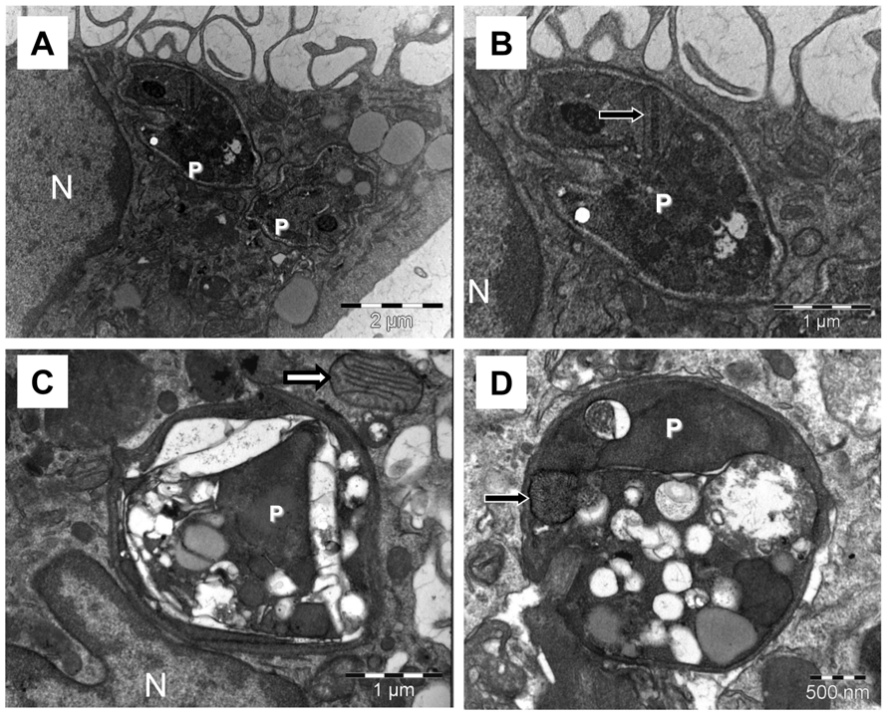
Oxidative damage of DETC is restricted to amastigotes in the phagosome. Transmission electron microscopy of human macrophages infected with *Leishmania amazonensis*. Human macrophages were infected with *Leishmania amazonensis* (5∶1 ratio) for 4 h and then treated with DETC (2 mM) for 10 h. Untreated macrophages displayed numerous well-preserved parasites (A and B). DETC-treated macrophages presented remarkably damaged parasites (C and D), whereas host cell and parasite mitochondria were not injured (white and black arrows, respectively). P indicates intracellular parasites and N indicates host cell nucleus, bars represent 2 µm (A), 1 µm (B and C) or 0.5 µm (D). Images are representative of 2 independent experiments.

Transmission electron microscopy was employed in order to document the mechanism of killing at the ultrastructural level of the cell at earlier time points, before parasite clearance. At 10 h of culture, control monolayers presented many well-preserved parasites (Fig. 3A and Fig. 3B), whereas DETC-treated cultures displayed remarkably damaged parasites, as evidenced by decreased electron-density (Fig. 3C and Fig. 3D). In cultures co-incubated with DETC and NAC, many parasites were observed with normal ultrastructure (data not shown), suggesting that DETC-induced killing occurs through oxidative damage. It is noteworthy that macrophage mitochondria were structurally unaffected, enforcing the hypothesis that DETC-triggered oxidative damage is phagosome-restricted.

### Selective toxicity towards mitochondria in DETC-treated *Leishmania* axenic promastigotes

**Figure 4:**
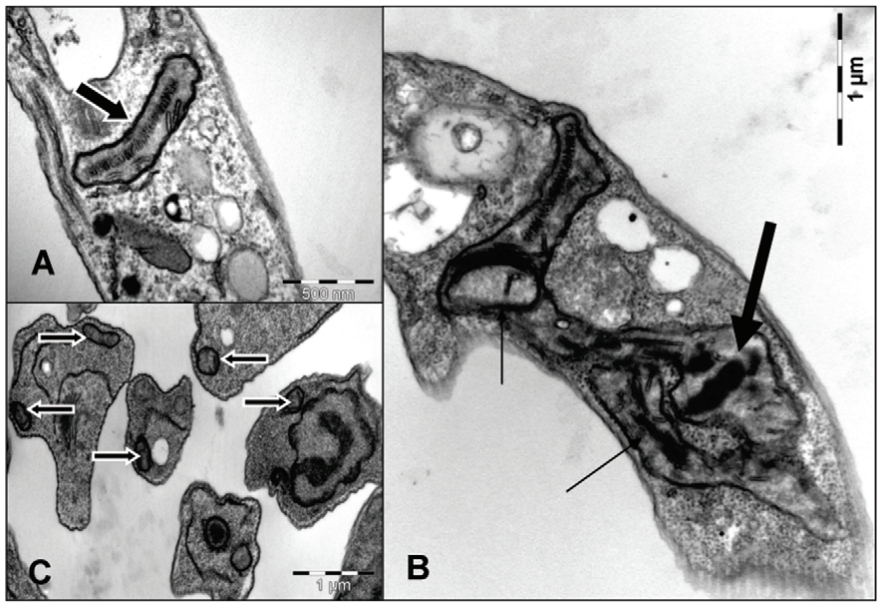
Oxidative damage of DETC in axenic promastigotes is restricted to mitochondria. Transmission electron microscopy of promastigotes form of *Leishmania amazonensis* in axenic culture treated with DETC. Promastigotes were treated with DETC (0.2 mM) for 1 h. (A) Untreated promastigotes displayed a well-preserved cytoplasm and electron-dense mitochondria with normal cristae. (B) DETC-treated promastigotes presented a well-preserved cytoplasm, besides enlarged mitochondria with reduced electron density, often with parallel or circular cristae (thin arrow). Dense core-containing mitochondria reacted positively for calcium oxalate (see Methods) by cytochemical detection (large arrow). (C) The use of NAC partially reverted the effects of DETC upon parasite mitochondria (large arrows with white margins). Images are representative of 2 independent experiments.

DETC is often used to inactivate mammalian intracellular copper-zinc superoxide dismutase, but might also affect *Leishmania* Fe-superoxide dismutase. We analyzed by transmission electron microscopy the action of DETC on *Leishmania amazonensis* axenic promastigotes *in vitro*. At 1 h of culture, DETC-treated parasites presented enlarged mitochondria with reduced electrondensity, often displaying parallel or circular cristae (Fig. 4B). DETC induced the formation of numerous dense core-containing cytoplasmic compartments which reacted positively for calcium oxalate (by cytochemical detection, see Methods) and therefore presumably comprise acidocalcisomes (Fig. 4B), as previously described [16]. DETC strongly inhibited parasite survival in axenic culture with an IC50 value of 0.16 mM (data not shown). The use of N-acetylcysteine (NAC), an anti-oxidant, was able to partially revert the effects of DETC, again suggesting a pro-oxidant effect of the drug upon the parasite (Fig. 4C), analogous to the host cell but at six-fold lower concentration.

### *In vitro* leishmanicidal effect of DETC in BALB/c murine macrophages

**Figure 5:**
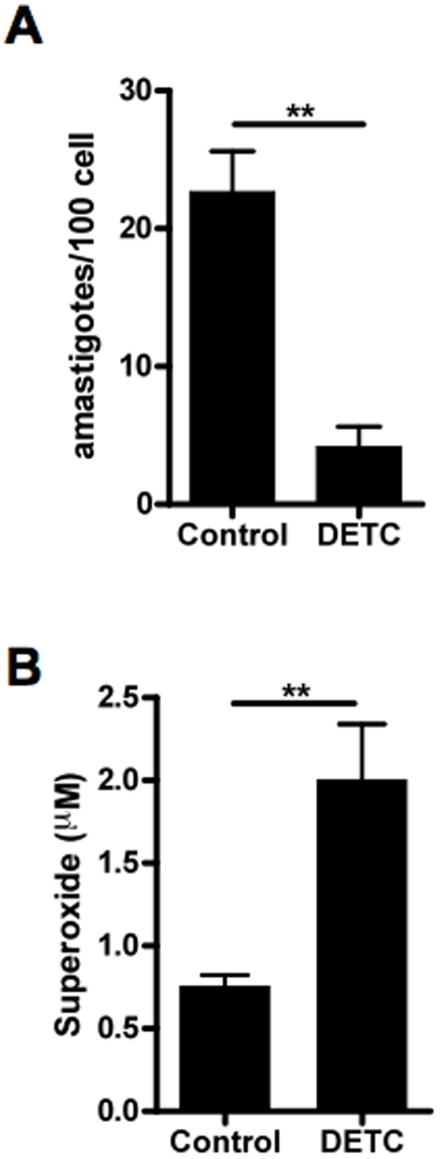
Leishmanicidal effect of DETC in murine macrophages *in vitro*. (A) Murine (BALB/c) macrophages were infected with *Leishmania braziliensis* (5∶1 ratio) and treated with DETC (2 mM). Number of intracellular amastigotes was quantified as described in Materials and Methods. Each bar represents the mean ± SEM of six (Mann Whitney test, **p = 0.0049). (B) Murine (BALB/c) macrophages were treated with DETC (2 mM) in the presence of hydroxylamine (0.5 mM) and supernatant was collected at 48 h. Superoxide production was quantified as described in Materials and Methods. Each bar represents the mean ± SEM of six (Mann Whitney test, **p = 0.0043).

Based upon the strong *in vitro* leishmanicidal effect of DETC in human macrophages, we decided to examine whether DETC might exert a leishmanicidal effect in a murine model *in vitro* and *in vivo*. Murine macrophages infected with *L. braziliensis in vitro* were treated with DETC as described above. DETC treatment induced a significant reduction in parasite load (Mann Whitney test, **p = 0.0049) (Fig. 5A), but to a lesser extent than observed in human macrophages (Fig. 1A, Fig. 1B and Fig. 1C), consistent with the modest superoxide production in murine macrophages (Mann Whitney test, **p = 0.0043) (Fig. 5B versus Fig. 2C). Similar results were obtained with *L. amazonensis* infection (data not shown).

### *In vivo* leishmanicidal effect of DETC in BALB/c mice challenged with *L. braziliensis*

**Figure 6:**
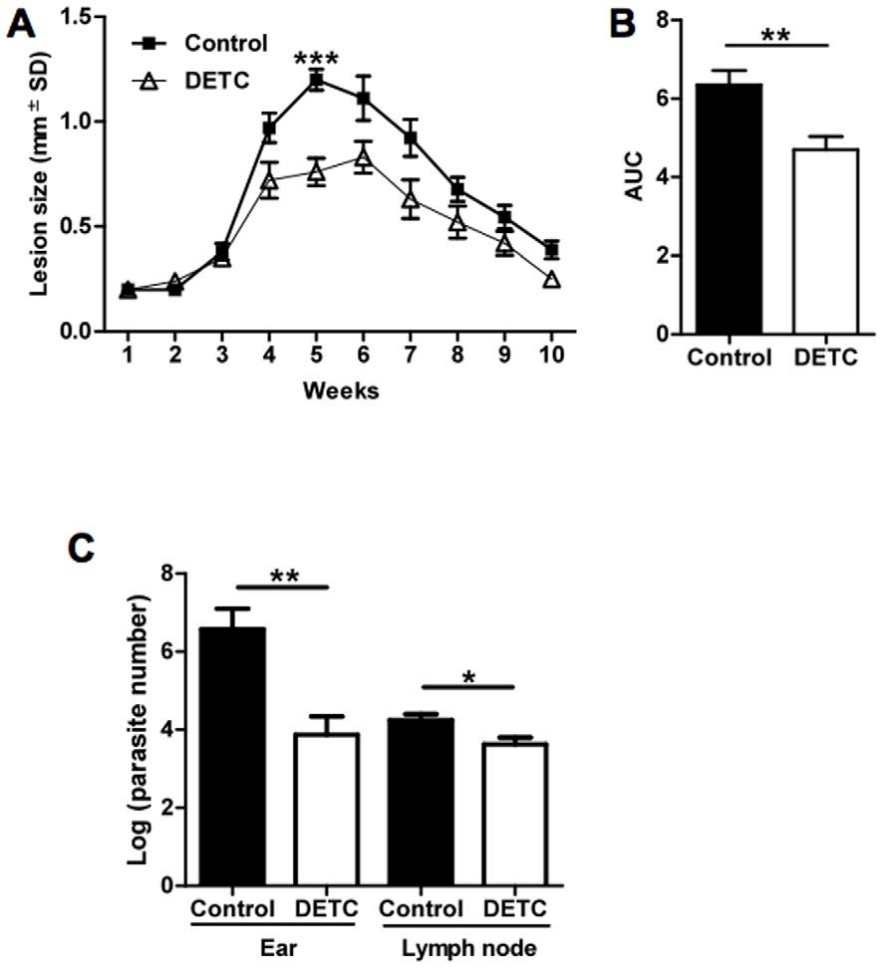
Leishmanicidal effect of DETC in BALB/c mice *in vivo*. Mice were infected with 10^5^ *L. braziliensis* promastigotes, and the course of lesion development was monitored for 10 weeks. (A) Lesion size (in millimeters) expressed as mean ± SEM of a representative experiment (n = 10 mice for each, control and DETC) (Unpaired *t* test, 5 weeks, ***p<0.0001). (B) Lesion size in mice challenged with *L. braziliensis* and treated with saline or DETC, as AUC (area under the curve) obtained in (A) for experimental (n = 10) and control mice (n = 10) were compared. Each bar represents the mean ± SEM of a representative experiment out of two (Unpaired *t* test, **p = 0.0041). (C) Parasite load of ear and draining lymph node were determined at 5 weeks post-infection via a limiting dilution assay. Ear parasite load data (normal distribution following log-transformation) represent the mean ± SEM (n = 6 each, control and DETC mice) of two experiments (Unpaired *t* test, **p = 0.0087). Lymph node parasite load data were not normally distributed, even after log-transformation, and are expressed as median ± SD (n = 6 each, control and DETC) (Mann Whitney test, *p = 0.032).

Using a recently established mouse model of cutaneous leishmaniasis that mimics human leishmaniasis (14), BALB/c mice were infected in the ear dermis with 10^5^ stationary phase promastigotes and lesion development and parasite burden were quantified. As previously described, parasite load could be determined in the ear dermis and draining lymph nodes of the animals at the second week post-infection (data not shown). Therefore, after two weeks of infection, animals were treated with DETC (50 mg/kg/day) or saline daily. The DETC group displayed significantly smaller lesions than the control group treated with saline, with a maximum peak difference at 5 weeks (Unpaired *t* test, ***p<0.0001) (Fig. 6A). In addition, the area under the curve (AUC) between the 2 groups was significant (Unpaired *t* test, **p = 0.0041) (Fig. 6B), demonstrating the sustained effect of DETC. To evaluate if lesion development was correlated with parasite multiplication at the site of infection, parasite load was quantified using a limiting dilution assay. Five weeks after infection, at the peak of lesion size in the control group, parasite load was 100-fold lower in the ear dermis of DETC group, when compared to control animals (Unpaired *t* test, **p = 0.0087) (Fig. 6C). In draining lymph nodes, parasite burden in the DETC group was 3.7 times lower than in the control group (Mann Whitney test, *p = 0.032) (Fig.6C). At 10 weeks following infection, parasites were no longer detectable in the ear dermis of both groups of animals, as previously described for this self-healing model [14].

## Discussion

In the present work, we demonstrated that DETC, a superoxide dismutase inhibitor, enhances parasite killing by macrophages *in vitro* and decreases lesion size and parasite load *in vivo*. In murine models of leishmaniasis, it is widely assumed that disease resistance is largely correlated with the expression of inducible NO synthase (iNOS) and NO production by infected cells [17]. iNOS deficient mice, as well as murine macrophages treated with iNOS inhibitor are highly susceptible to *Leishmania spp.* [18]–[20]. On the other hand, the leishmanicidal role of superoxide anion in murine leishmaniasis *in vivo* has been demonstrated to be important only in the earlier stages of infection [18]–[19]. However, macrophages derived from NADPH oxidase (nicotinamide adenine dinucleotide phosphate-oxidase)-deficient and wild type mice had equal *in vitro* capacity to kill intracellular *Leishmania spp.* (18–19). Nevertheless, our group has recently shown that NAC (a precursor of glutathione, a powerful antioxidant) by itself was able to increase parasite burden in murine macrophages infected with *L. braziliensis in vitro* [20]. In addition, the use of a superoxide scavenger (Tempol) significantly inhibited leishmanicidal activity in wild type IFN-γ-treated murine macrophages [21]. Conversely, NAC treatment *in vivo* induced parasite killing, independent of the *Leishmania spp.* or mice background [22]–[24]. This unexpected effect of NAC *in vivo* might be explained by the fact that glutathione was able to induce NO production in murine macrophages *in vitro* [25]. Our data regarding superoxide-mediated parasite killing (11, this manuscript) do not challenge the consensus in the literature that nitrogen-derived free radicals are the main leishmanicidal effectors in murine models. Rather, we propose that NO-independent, superoxide-dependent parasite killing does occur under specific conditions, such as DETC treatment. In agreement with this hypothesis, DETC treatment was unable to induce NO production, both *in vitro* (supernatants of DETC-treated macrophages, data not shown) and *in vivo* (sera of DETC-treated mice, data not shown), inspite of its significant leishmanicidal effect (Fig. 4 and 5).

In human macrophages, we have recently demonstrated a NO-independent, superoxide-dependent leishmanicidal mechanism [11]. In this study, our data confirmed the capacity of infected human macrophages to produce high amounts of superoxide, as compared to infected murine macrophages. Despite the multiple possible mechanisms of action of DETC [26]–[27], our results support the hypothesis that DETC *in vitro* functions through an increase of superoxide anions release, since DETC-induced parasite killing in human macrophages is, at least partially, reverted by the addition of NAC. Inducing superoxide production in human macrophages through SOD inhibition appears to be a valuable therapeutic alternative, considering current first-line treatment of leishmaniasis with pentavalent antimonials is associated with significant toxicity and worldwide increasing resistance [4], [6], [8]–[9]. DETC has already been used *in vivo* as an adjuvant of the immune system (also known as Imuthiol or Dithiocarb), including in HIV-1-infected patients, leading to a significant delay in progression to AIDS, as well as minimizing opportunistic infections [28]. In addition, it has been shown that monocytes from healthy volunteers injected with DETC displayed increased *in vitro* microbicidal activity against *Mycobacterium tuberculosis* [29].

Interestingly, the effect of DETC upon *Leishmania* promastigotes suggests that leishmanial SOD plays a role in parasite redox balance not only in intramacrophage parasites [30], but also detoxifies endogenous superoxide production in axenic forms. In fact, *Leishmania* mitochondria produce superoxide and parasite iron superoxide dismutase localises to this organelle in *L. donovani* [31]. Besides, DETC can also form a complex with Fe^2+^ [32], which might lead to inhibition of enzyme activity of iron superoxide dismutase in *Leishmania spp.*. Both axenic *Trypanosoma cruzi* epimastigotes [33] and *L. amazonensis* promastigotes [34] have been shown to undergo severe mitochondrial injury, including circular cristae, in oxidative stress conditions. Different leishmanicidal compounds induce the formation of acidocalcisome-like compartments, which may represent autophagic vacuole residual bodies [35]. Despite early ultrastructural evidence [36]–[37], it was recognized only recently that acidocalcisomes may be derived from lysosome-related multivesicular bodies [16]. However, in intracellular amastigotes, DETC induces marked ultrastructural differences, since parasite mitochondria appear preserved but the entire parasite appears extremely vacuolised in treated macrophages (Fig. 3D, black arrow).

Due to its pronounced effect upon parasite survival, either as intracellular amastigotes, or as extracellular promastigotes and because of the absence of *in vitro* [38] or *in vivo* [39] toxicity, DETC can be considered as an alternative therapy in those forms of leishmaniasis with higher morbidity and mortality. Most likely candidates are diffuse cutaneous leishmaniasis patients with nonhealing lesions, which are often refractory to standard antimonial treatment [7]. Interestingly, we have demonstrated that SOD treatment led to strong increase in parasite burden [11] and the appearance of large parasitophorous vacuoles (Khouri *et al.*, submitted), reminiscent of typical nodular lesions in diffuse leishmaniasis [40]. Finally, DETC therapy might also be envisaged in visceral leishmaniasis, considering the higher morbidity/mortality and more frequent therapeutic complications of, this clinical form, especially in the case of HIV co-infection, which is of increasing epidemiological importance in Southern Europe and Latin America [1]–[4].
